# A Web-Based Model to Predict a Neurological Disorder Using ANN

**DOI:** 10.3390/healthcare10081474

**Published:** 2022-08-05

**Authors:** Abdulwahab Ali Almazroi, Hitham Alamin, Radhakrishnan Sujatha, Noor Zaman Jhanjhi

**Affiliations:** 1 Department of Information Technology, College of Computing and Information Technology at Khulais, University of Jeddah, Jeddah 21959, Saudi Arabia; 2 School of Information Technology & Engineering, Vellore Institute of Technology, Vellore 632001, India; 3 School of Computer Science (SCS), Subang Jaya 47500, Malaysia

**Keywords:** brain disorder, dementia, data imputation, scaled conjugate gradient, performance measures

## Abstract

Dementia is a condition in which cognitive ability deteriorates beyond what can be anticipated with natural ageing. Characteristically it is recurring and deteriorates gradually with time affecting a person’s ability to remember, think logically, to move about, to learn, and to speak just to name a few. A decline in a person’s ability to control emotions or to be social can result in demotivation which can severely affect the brain’s ability to perform optimally. One of the main causes of reliance and disability among older people worldwide is dementia. Often it is misunderstood which results in people not accepting it causing a delay in treatment. In this research, the data imputation process, and an artificial neural network (ANN), will be established to predict the impact of dementia. based on the considered dataset. The scaled conjugate gradient algorithm (SCG) is employed as a training algorithm. Cross-entropy error rates are so minimal, showing an accuracy of 95%, 85.7% and 89.3% for training, validation, and test. The area under receiver operating characteristic (ROC) curve (AUC) is generated for all phases. A Web-based interface is built to get the values and make predictions.

## 1. Introduction

Worldwide there are over 55 million individuals suffering from dementia in 2020. The number of individuals who have dementia almost doubles every 20 years. Approximately 60% of people affected by dementia live in middle- and low-income countries. There are around 10 million new cases every year worldwide, which implies one new case every 3 s. The worldwide annual cost including all direct and indirect services is above USD 1.3 trillion dollars. This means that if we consider dementia as a country, its economy would be the fourteenth largest economy globally. It can also be observed from the world Alzheimer’s report that approximately three-quarters of cases are not diagnosed [1].

Patients, their family and their caregivers are all affected by dementia. While a cure for any type of dementia is improbable soon, existing symptomatic treatments offer hope for improving patients’ and quality of life of caregivers. The neurodegenerative disease called Alzheimer’s disease is indicated by a progressive amyloid build-up of intracellular neurofibrils and extracellular plaques. Currently, no disease-modifying treatments are available; however, several drugs that can help with cognition are available [2,3].

Dementia is a set of symptoms that significantly damage memory, social and reasoning skills when they considerably affect regular functioning. Dementia is caused by multiple illnesses. The signs and symptoms of dementia vary depending on the underlying cause, but frequent ones include cognitive and physiological changes. Dementia has a wide range of main neurologic, neuropsychiatric and medicinal causes. It is typical for a patient’s dementia syndrome to be caused by a number of illnesses [4,5].

**Figure 1:**
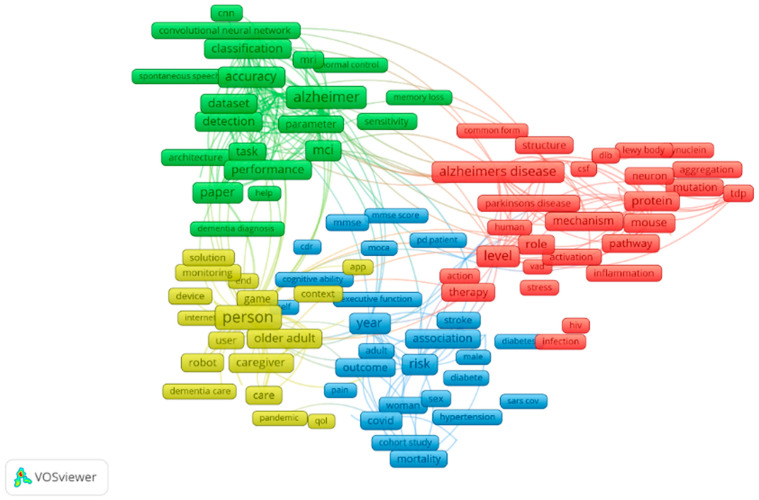
Bibliometric network for dementia.

Quantum research work carried on in the field of dementia with the subject areas of computer science, decision sciences and multidisciplinary is queried in Scopus. Based on the retrieved data, a bibliometric network is drafted using VOSviewer. Text mining functionality is employed to obtain a co-occurrence network relating relevant terms that were retrieved from the amount of scientific literature. Visualising the same provides the inference that classification, convolutional neural network, Alzheimer’s disease and so on have high contributions in this field, and it is illustrated in Figure 1 [6].

For the contribution of the work, we have designed a model using the SCG algorithm that can classify patients as demented, nondemented and converted and using this dataset, previously regular machine learning training was only carried out. Using a non-imagery dataset to get a high accuracy model average imputation is incorporated. The bibliometric network diagram shows the impact of the various research works carried out in this domain. Based on the experimental performance to track dementia, it shows an accuracy of 95%, 85.7% and 89.3% for training, validating and testing. Ideal ROC curves are generated for all phases.

The work is carried out in sequence. Section 2 of the paper briefs the recent works using the SCG algorithm in various verticals. Section 3 provides the data visualisation and details the entire work with an illustration. Section 4 elaborates on the investigational argument, followed by a comparison with recent outcomes, conclusions and future work are presented in Section 5.

## 2. Related Work

The SCG is a supervised learning technique. Basic back-propagation algorithm (BP), one-step Broyden–Fletcher–Goldfarb–Shanno (BFGS) memoryless quasi-Newton technique and conjugate gradient line search algorithm (CGL) and are all benchmarked against SCG. The SCG algorithm is automated, does not have any parameters that are user-dependent, and removes the line search which is exhausting that BFGS and CGL utilise to establish a appropriate size of step in each iteration. The SCG algorithm is significantly swifter than BFGS, BP and CGL, according to tests [7]. Applications of SCG in the case of supervised datasets are more irrespective of the type of data. Because of its wide variation, character recognition and handwritten text are more difficult than handwritten numeric and computer-printed text recognition. Neural network-based approaches provide the most trustworthy results in text and handwritten character recognition. However, it is dependent on several important factors such as the number of samples used in training, features that are reliable and the number of features in each character, the time required for training and the handwriting variations. Various important handwriting characteristics are gathered and provided to neural networks to perform training [8].

Robot selection models used in industries are complicated nonlinear systems which are solved by employing vigorous estimating techniques such as neural network algorithms. The use of a pattern classification technique for neural networks to find manipulator properties, including quantitative and qualitative traits, is proposed in this paper. Various training functions which can update bias and weight values are used in training a feedforward neural network. The application of SCG as a training algorithm for robot selection prediction techniques is investigated in this paper [9]. Based on ultrasonic signal energy, this research offers an autonomous system driven by data for fatigue damage detection and associated mechanical structure damage risk classification. The fundamental notion depends on the attenuation of signal and attenuation process stability. The attenuation represents resistance to fatigue damage increase, whereas the stability represents information for damage quantification. The SCG back-propagation approach was used to train the suggested neural network (NN) model. Damage detection and classification into five classes of increasing danger are possible with the NN model [10].

The price of fuel determines the growth of any country. Fuel price fluctuations have both direct and indirect economic consequences. Because of the greater degree of inflation in the oil business, a nation’s economy will be limited. The neural networks are trained using three different training algorithms: Levenberg–Marquardt, SCG and Bayesian regularisation [11]. The location of the mine pit will be the sub-groundwater level as mining depth increases. The intrusion of groundwater into pits raises costs, diminishes productivity and compromises worker safety. The prediction of water table levels is extremely valuable for water resource management in mining. For optimization, SCG is utilised [12].

This research aims to predict eucalyptus productivity from data comprising silviculture, biotic and abiotic data by developing an efficient ANN. The time required for processing to provide a solution which is accurate was characterised as efficiency [13]. The objective of this research is to anticipate stock market closing prices in India using ANNs. The literature identified some macroeconomic indicators and a few stock market factors of markets worldwide as input variables. The Bombay Stock Exchange (BSE) Sensex is forecasted using an ANN using SCG [14]. An ANN method was used to estimate landslide risk in this study. The landslides resulting due to factors that affect temporal and spatial distribution can be mitigated by this research work [15].

An artificial neural network is the highly utilised machine-learning practices for forecasting software components that are defect-prone. In this work, a system based on a cloud for software fault prediction in real time is given [16]. For the first time, models estimating formation damage accurately in terms of permeability of damage during a water flooding operation were developed as relatively innovative intelligent models [17]. Perceptron of multiple layers and linear stepwise regression have been proposed for grading agarwood oil. The stepwise regression’s output features were used as input features in a multilayer perceptron network to model agarwood oil [18].

There are two major flaws with the existing table tennis robot system. One is the pace of the ball used in table tennis, which moves quickly and makes it tough for the system to react soon. The second issue is that the robot cannot distinguish ball movement types, such as wait, top rotation, rotation, no rotation and so on. This issue is addressed by presenting a tracking method to track the target trajectory that incorporates the machine’s vision [19]. The study’s goal is visualising how various parameters affect diabetes data to predict diabetic patients. For an effective forecast of diabetes, this research develops an improved ANN model trained using SCG [20].

Utilising a wavelet transform of continuous type and derivative filter, this paper provides a unique method for detecting myocardial infraction and block of bundle branch by feeding in ECG signal obtained from multiple lead ECG devices. Below 50 Hz frequency signal was obtained, and a filter based on derivative was applied for the features extraction process. BBB and MI signals were also subjected to continuous wavelet transformations. To acquire the features, the coefficients of CWT were mined, and then signals were recreated from the wavelet. By adding these characteristics to the classifier, the derivative-based filter and CWT outputs were assessed [21]. Recent advancements in computer networks resulted in network security related issues in smart cities. The fundamental goal of this research is to classify the genuineness of packets, and soft computing has been used to do so; moreover, to integrate fuzzy logic systems into analysis and adaptive capabilities to prevent intrusion [22].

This research aims to develop a device that can measure total body water using an ultrasonic sensor, a load, and a bio-electric impedance analyser to calculate an individual’s total body water level. During neural network training, several hidden neurons were utilised and compared, and it was discovered that using 10 neurons provided the best Pearson’s correlation value and lowest mean square error. According to the results, the SCG algorithm performs better with 10 neurons [23]. Developers and researchers of the twenty-first century have a considerable focus on harvesting solar energy in an optimal way. Solar energy optimization is purely dependent on the amount of sunlight that reaches the solar panels. Radiation can be measured using a variety of equipment and calculated using a variety of estimating algorithms. The feed-forward neural network with back-propagation and a neural network of three-layer with one-layer hidden is deployed [24].

## 3. Proposed Methodology

**Figure 2:**
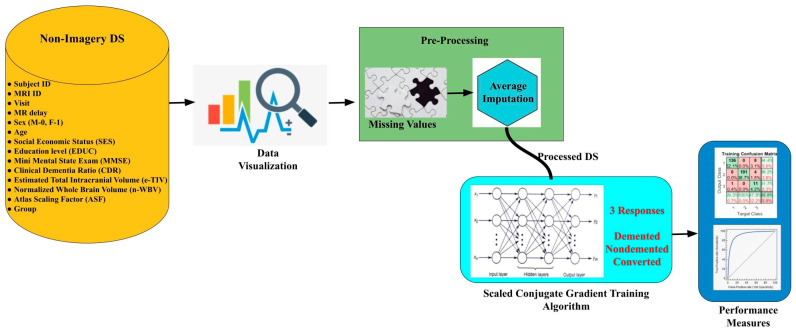
Entire workflow.

Working with the medical dataset is a challenging task, and initially, we need to acquire domain knowledge to get insights into the requirement [25,26]. Its mandate is to visualize, analyse and pre-process based on nature. Missing data is required to be cared for a lot to build a better performance system. Based on the distribution of data, a suitable approach can be utilised. Figure 2 represents the entire workflow, choosing dataset, data visualisation and data pre-processing to handle missing values using average imputation, followed by SCG. The ANN concept is used in making a good prediction model.

### 3.1. Non-Imagery Datasets

**Figure 3:**
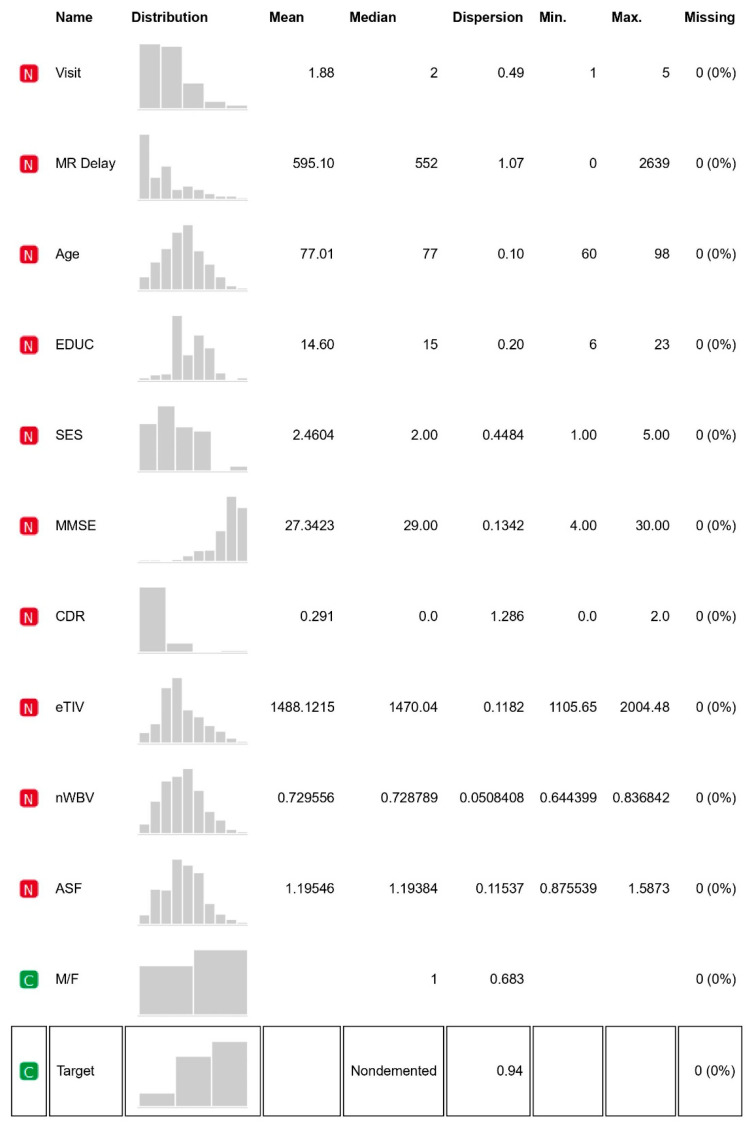
Feature statistics.

The OASIS data set includes MRI data of demented and nondemented right-handed (R) people of ages ranging between 60 and 96. With 373 MR sessions, a sample size of 150 women and men appeared in scanning sessions for three or more visits; there was at least one-year of separation between each session. Figure 3 provides the feature statistics of the considered datasets and along with it other features meant for identification are right-hand indications, subject ID and MRI ID. Its non-imagery datasets comprise socio-demographic—M/F (male, female) gender, age (60–98 years), education level (EDUC), social-economic status (SES) and clinical features—Mini-Mental State Exam (MMSE), Atlas Scaling Factor (ASF estimated total), intracranial volume (e-TIV), clinical dementia ratio (CDR), normalised whole brain volume (n-WBV) and delay pertaining with brain development as per magnetic resonance image (MR Delay). As per the statistics, MMSE and SES have missing values. Group is the target feature classifying the record into nondemented, converted and demented, based on the values of the features it categorises [27,28].

**Figure 4:**
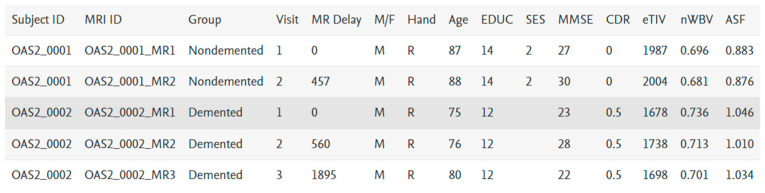
Sample data.

The values of the features as per the original are shown in Figure 4. For the experimental working, attribute M/F, male and female mapped as 0 and 1, respectively, Hand is R in all the records, so we left it, similarly for the target attribute group, nondemented, demented and converted mapped to 3, 2 and 1, respectively. Other features are numerical.

### 3.2. Data Visualization

It helps to know about the relationship of various features that contribute to the class value. Figure 3 mentions the statistics of each variable with clarity. It is evident that MMSE and SES have some missing values that need to be filled.

**Figure 5:**
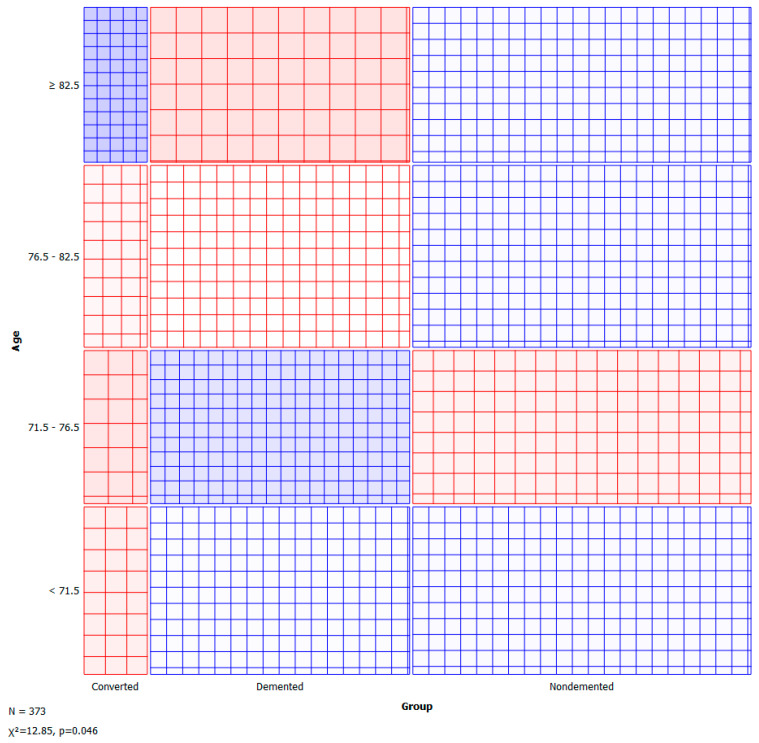
Sieve—group vs. age.

**Figure 6:**
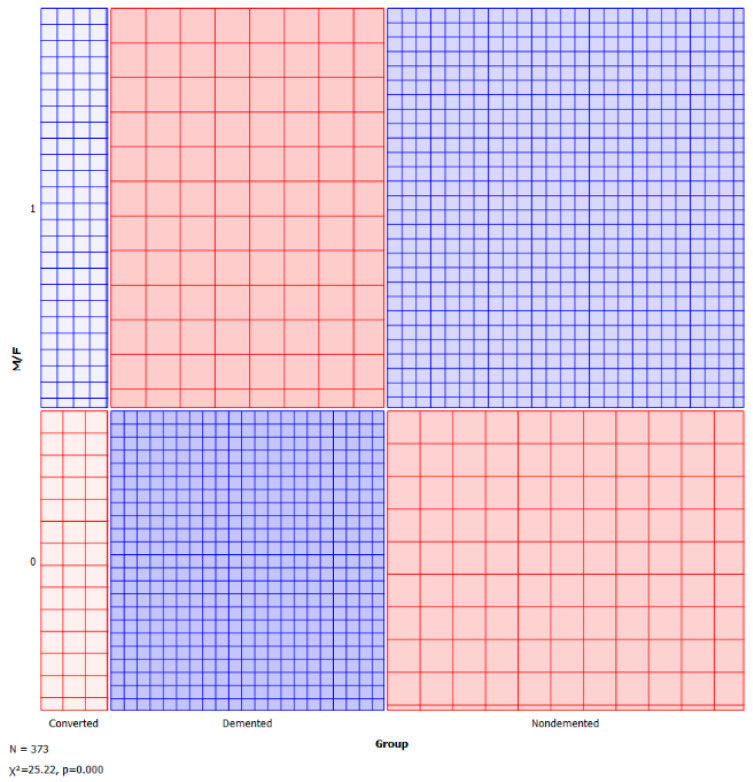
Sieve—group vs. M/F.

A sieve diagram is a graphical approach for visualising and comparing the frequencies in a contingency table in two ways to the expected frequencies under independence assumptions. Riedwyl and Schüpbach proposed the sieve diagram in a technical report in 1983, and it was later dubbed a parquet diagram. The area of every rectangle in the graph is proportional to the expected frequency. The number of squares present in every rectangle represents the observed frequency. The shading density represents the predicted frequency and observed frequency difference (proportional to standard Pearson residual), with colour indicating that the deviation is negative (red) or positive (blue). Figure 5 and Figure 6 visualise the pattern of association between group vs. age and M/F.

### 3.3. Pre-Processing—Missing & Average Imputation

Data pre-processing helps in improving results. Missing values are required to be handled by proper analysis. Imputation of the dataset can be achieved by a couple of methods. Based on the size of datasets and the percentage of missing values, our work utilised the average imputation approach. It is used in the numerical values column and computing the average value in the missing place. Obviously, the values are filled based on the corresponding column values. By varying methods, average imputation has been found to serve better results [29,30].

### 3.4. SCG

**Figure 7:**
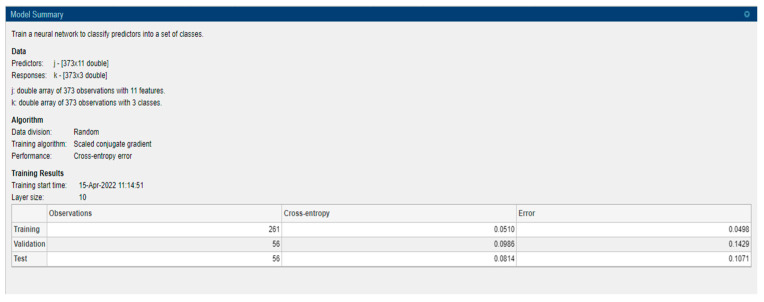
Model summary.

A learning algorithm which is a supervised type is employed to feed-forward neural networks. It chooses the step size and searches the direction more effectively by looking into the information of second-order approximation. SCG algorithm integrates the conjugate gradient approach and model-trust region approach. It was developed by Moller. Even though the algorithm depends on the direction of the conjugate, unlike other algorithms, it does not execute a line search at every step. This property of not performing a line search at every iteration makes it less time-consuming and computationally less expensive [30,31]. It consists mainly of neurons that represent input and output variables and layers that are intermediate coupled by weights. The method employed, count of neurons available in the hidden layer, member function type and learning rate all influence the performance of ANNs. A model summary is provided in Figure 7. The number of hidden neurons is 10 and it is based on 2/3 of the input layer summing with the number of output layers.

In MATLAB, the SCG method can be adopted by using the ‘trainscg’ function command which apprises the values of bias and weight. To train a model using SCG, its weight, transfer and net input functions should be derivative functions. The size of step is quadratic approximation function which makes SCG independent of user-defined parameters and more robust. Points in weight spaces and steepest descent vectors are determined recursively from the conjugate system and weight space points, respectively. In every iteration, the same algorithm is applied to find the global error function. As the function describing the error is non-quadratic, the algorithm does not meet in N steps necessarily. If it does not converge in N steps, it is restarted. This implies that error should be a quadratic function [32].

## 4. Experimental Results and Discussions

Data comprise 373 records with 11 predictors and 3 responses, namely demented, nondemented and converted. Data division is performed randomly with an artificial neural network based on the SCG algorithm and cross-entropy error performance with 10 as layer size. For model building 70% of data is considered as training, 15% considered as validation and 15% as testing.

### 4.1. Cross-Entropy

**Figure 8:**
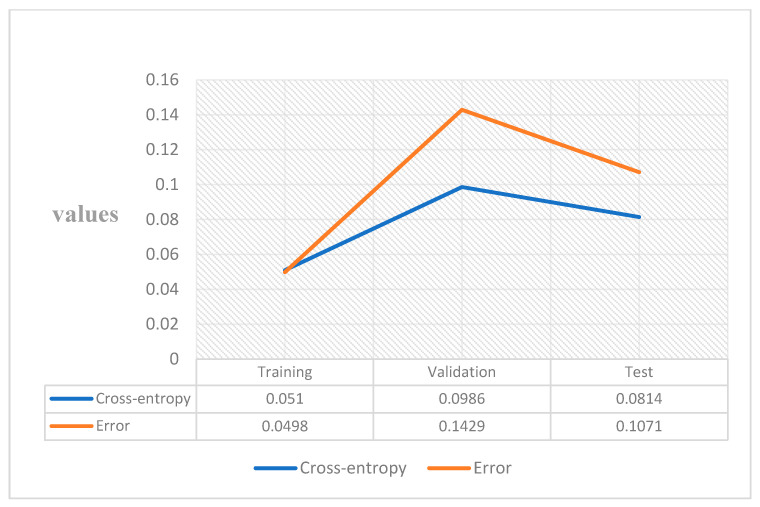
Cross-entropy and error.

A widely used loss function for optimisation of models that perform classification is cross-entropy. It is important when dealing with numerous classes and trying to get our model to converge faster by reducing loss. The performance of a model performing classification whose output is between 0 and 1 representing probability is measured in terms of cross-entropy loss, which is also called log loss. Due to the difference between the actual label and projected likelihood, log loss grows. Cross-entropy loss of an ideal model should be zero. Cross-entropy < 0.05 is considered on the right track and <0.2 is considered fine. In the case of training, our model is on track and is fine for the validation and test part [33]. In machine learning, the error is used to examine how effectively our model can predict new data as well as data it has not seen before. We select the machine learning model that performs best for a given dataset based on our error. The error essentially represents how well your network performs on a (training/testing/validation) set. Figure 8 shows the value, and it is low. A low error is desirable, while a high error is unquestionably undesirable.

### 4.2. Best Validation Performance

**Figure 9:**
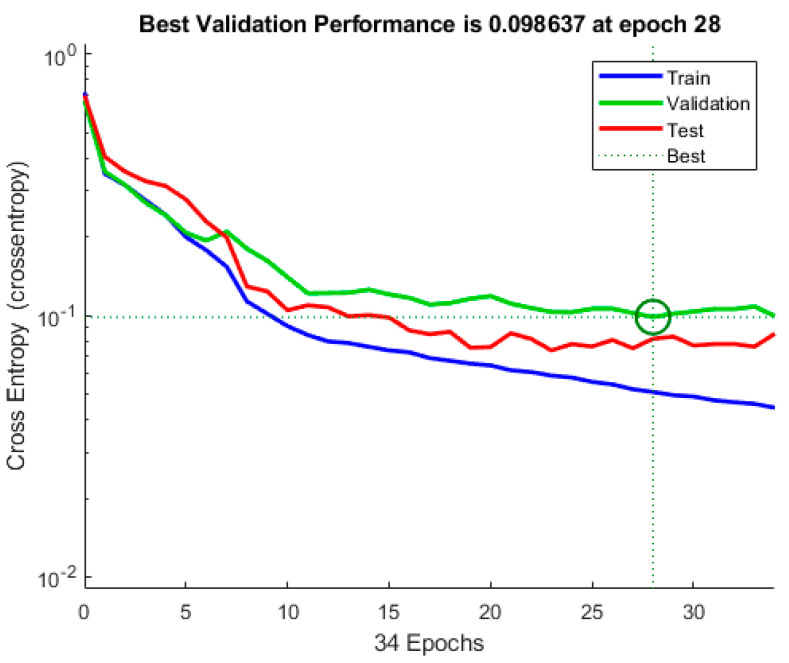
Validation performance.

The cross-entropy of a training ANN model, validation ANN model (check) and testing ANN model are shown in Figure 9. According to this graph, the validation step with the least cross-entropy occurs at epoch 28, with the best validation performance of 0.098637. It is worth noticing that training continues till the network’s cross-entropy reduces for the vector of validation. Furthermore, the analysis stops at 30, i.e., after the best performance of validation epoch 28, there are two mistake repeats.

### 4.3. AUC

**Figure 10:**
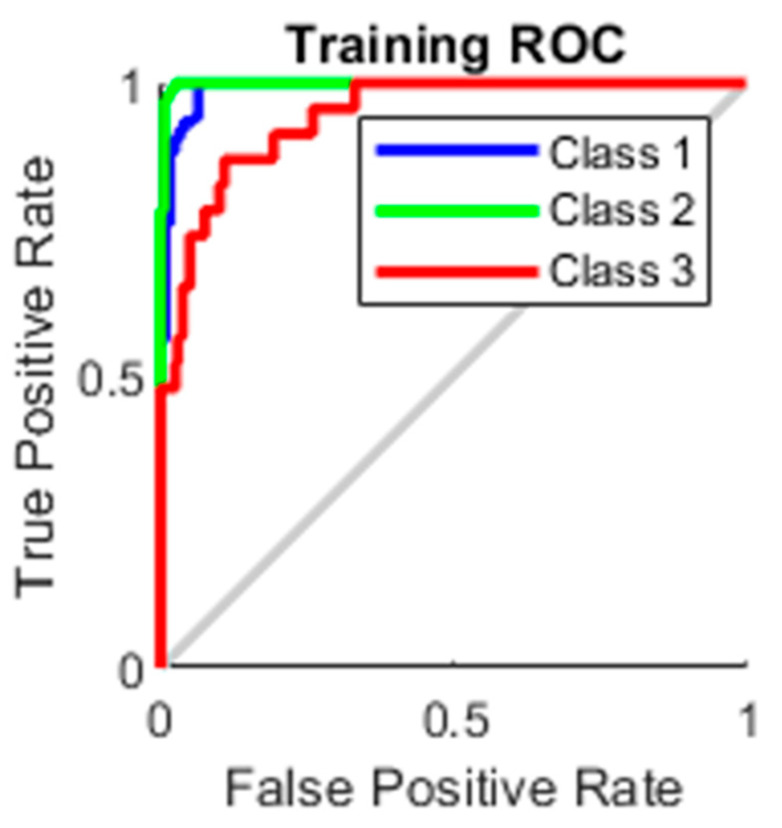
Training ROC.

**Figure 11:**
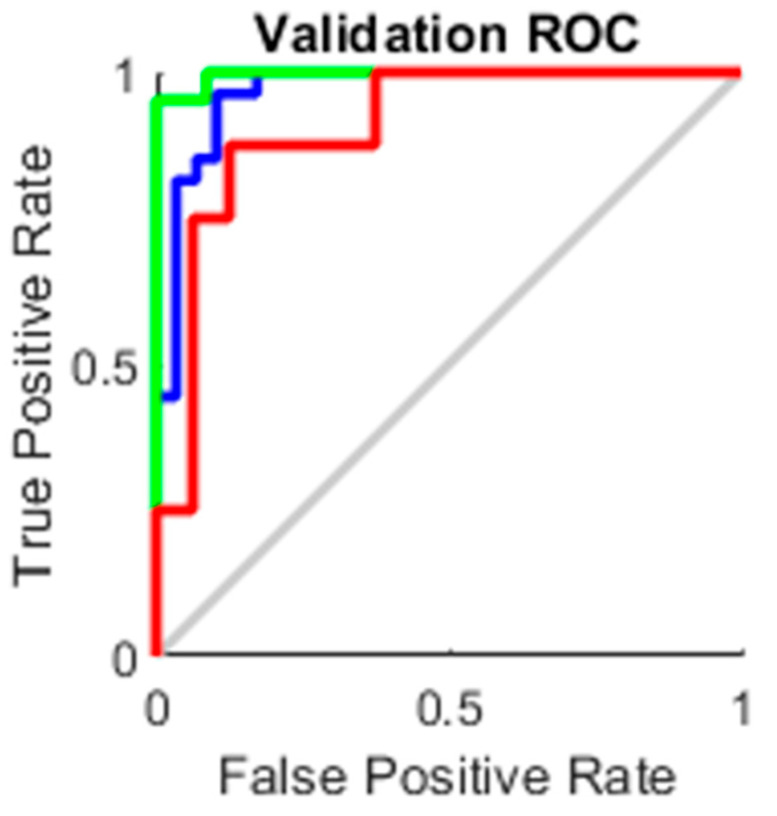
Validation ROC.

**Figure 12:**
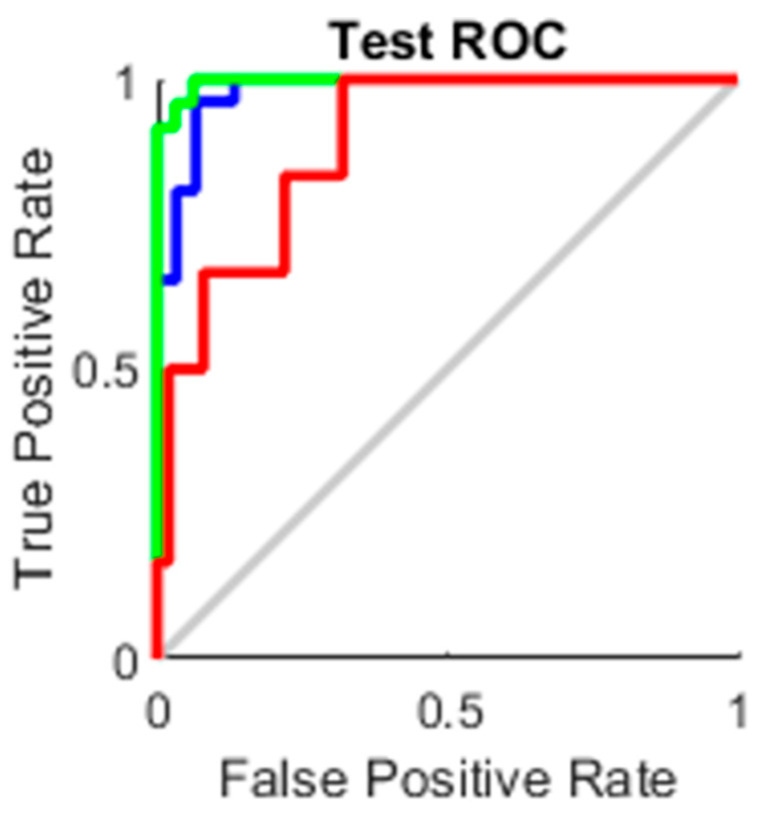
Test ROC.

**Figure 13:**
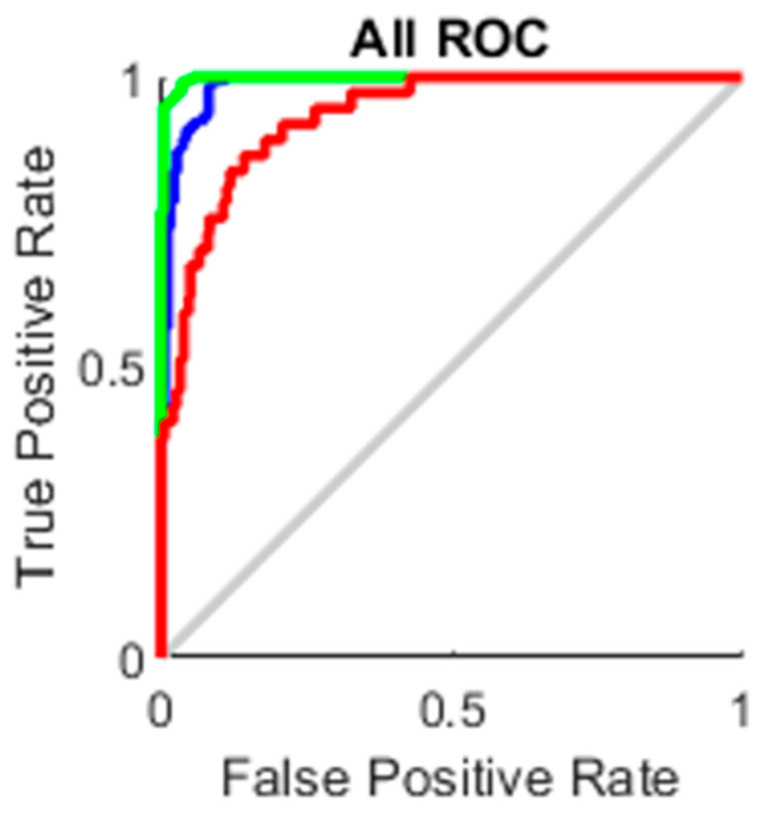
All ROC.

The true-positive rate opposed to the false-positive rate across various cut-offs represented by a plot produces a receiver operating characteristic curve. In “ROC space,” the ROC curves corresponding to increasing the diagnostic test’s discriminant capacity are gradually nearer to the top left corner. The idea of a “separator” (or choice) variable underpins the concept of a ROC curve. If the “criterion” or “cut-off” for positivity on the decision axis is changed, the rates of positive and negative diagnostic test findings will alter. Rather than relying on a single operating point, the AUC represents the full position of the ROC curve. The AUC is a useful and combined sensitivity and specificity measure that describes. Figure 10, Figure 11, Figure 12 and Figure 13 represent the training, validation, testing and overall ROC for the diagnostic model, and its AUC value of 0.9 to 1 is considered excellent, and 0.8 to 0.9 is considered good. In this work, the illustration clearly shows excellent and good results [34,35].

### 4.4. Confusion Matrix (CM)

The predicted class (output) is represented by row while the true class on the CM diagram (target) is represented by column. Table 1, Table 2, Table 3 and Table 4 represent the training, validation, testing and overall CM for the diagnostic model. Sloping cells relate to classified observations, respectively. The off-slope cells represent observations that were inaccurately categorised [36]. Every cell shows the count of observations and the percentage of the total number of observations. In the case of training, precision for 3 classes is 94.4%, 96.2% and 91.7%, and recall for 3 classes is 99.3%, 100% and 47.8% with an accuracy of 95%. In the case of validation, precision for 3 classes is 86.7%, 83.3% and 100%, and recall for 3 classes is 96.3%, 95.2% and 25% with an accuracy of 85.7%. Similarly, the test and all criteria created high results.

### 4.5. Web Interface

**Figure 14:**
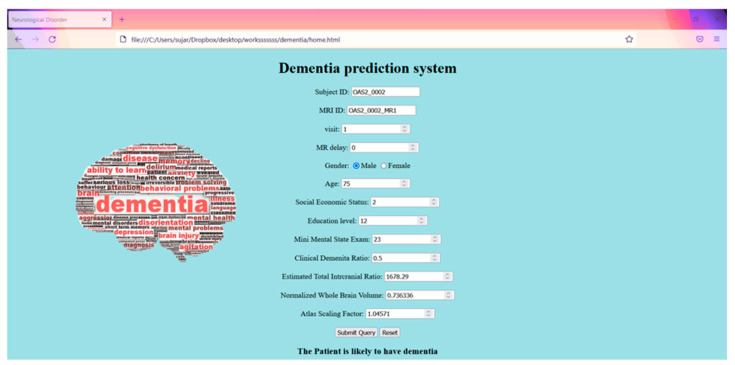
Web interface.

A web-based interface is built to ensure fast decision making by keying in the values. Figure 14 is a screenshot of the dementia prediction system.

## 5. Conclusions and Future Work

In comparison with existing works carried out with the dementia dataset employing a support vector machine, accuracy and precision of 68.75% and 64.18% were shown [37]. However, in our model, average imputation is used in the pre-processing data stage to get rid of missing values followed by the SCG to build a reliable model and, in turn, help in perfect prediction. The discussed model attained an accuracy of 95%, 85.7% and 89.3% for training, validation, and test and overall, 92.8% is achieved. An ideal ROC curve is generated for all phases. Cross-entropy and error are minimum. As mentioned in the contribution, we have built a web-based model with the SCG approach and average imputation used to handle missing values. It is a reliable model to make better predictions.

Dementia cases keep on increasing across the sphere due to lifestyle and as of now, it is an incurable ailment. From a biotechnology point of view, blood biomarkers are under research to predict dementia before its onset. Thoroughly analysing the existing data from various medical offices and pruning data correctly with the help of a bio-inspired algorithm will optimise the diagnosis of the disease at the earliest. With a hefty dataset, deep learning could be used to get more correlations to make an earlier diagnosis.

## Figures and Tables

**Table 1:** Training CM.

	Training Confusion Matrix			
Output Class	136 52.1%	0 0.0%	8 3.1%	94.4% 5.6%
	0 0.0%	101 38.7%	4 1.5%	96.2% 3.8%
	1 0.4%	0 0.0%	11 4.2%	91.7% 8.3%
	99.3% 0.7%	100% 0.0%	47.8% 52.2%	95.0% 5.0%
	1	2	3	
	Target Class			

**Table 2:** Validation CM.

	Validation Confusion Matrix			
Output Class	26 46.4%	1 1.8%	3 5.4%	86.7% 13.3%
	1 1.8%	20 35.7%	3 5.4%	83.3% 16.7%
	0 0.0%	0 0.0%	2 3.6%	100% 0.0%
	96.3% 3.7%	95.2% 4.8%	25.0% 75.0%	85.7% 14.3%
	1	2	3	
	Target Class			

**Table 3:** Test CM.

	Test Confusion Matrix			
Output Class	26 46.4%	1 1.8%	3 5.4%	86.7% 13.3%
	0 0.0%	22 39.3%	1 1.8%	95.7% 4.3%
	0 0.0%	1 1.8%	2 3.6%	66.7% 33.3%
	100% 0.0%	91.7% 8.3%	33.3% 66.7%	89.3% 10.7%
	1	2	3	
	Target Class			

**Table 4:** All CM.

	All Confusion Matrix			
Output Class	188 50.4%	2 0.5%	14 3.8%	92.2% 7.8%
	1 0.3%	143 38.3%	8 2.1%	94.1% 5.9%
	1 0.3%	1 0.3%	15 4.0%	88.2% 11.8%
	98.9% 1.1%	97.9% 2.1%	40.5% 59.5%	92.8% 7.2%
	1	2	3	
	Target Class			

## Data Availability

On request.
